# The Fears and Hopes of Ukrainian Migrant Workers in Poland in the Pandemic Era

**DOI:** 10.1007/s12134-023-01051-7

**Published:** 2023-05-25

**Authors:** Olena Shelest-Szumilas, Marcin Wozniak

**Affiliations:** 1 Department of Education and Personnel Development, Poznan University of Economics and Business, Poznan, Poland; 2 Adam Mickiewicz University, Poznań, Poland

**Keywords:** COVID-19, Labor Market, Sentiment Analysis, Ukrainian Immigrants in Poland, Topic Extraction

## Abstract

Due to the COVID-19 pandemic, many immigrants found themselves in extremely unstable situations. The recent contributions show that employment decline in the first several months of the lockdown was higher for migrant workers than for natives. At the same time, migrants were less likely to find new employment in the recovery months. Such circumstances may result in an increased level of anxiety about one’s economic situation. On the other hand, an unfavorable environment may induce resources that could help to overcome it. The paper aims to reveal migrants’ concerns together with ambitions connected with the economic activity during the pandemic. The study is based on 30 individual in-depth interviews with Ukrainian migrant workers from Poland. The research approach was based on Natural Language Processing techniques. We employed sentiment analysis algorithms, and on a basis of selected lexicons, we extracted fears and hopes that appear in migrants’ narrations. We also identified major topics and associated them with specific sentiments. Pandemic induced several matters connected with e.g., the stability of employment, discrimination, relationships, family, and financial situation. These affairs are usually connected on the basis of a cause-and-effect relationship. In addition, while several topics were common for both male and female participants, some of them were specific for each group.

## Introduction

Labor markets in many countries have suffered from the effects of the COVID-19 pandemic. Several hundred thousand workers have lost their jobs because of the economic slowdown and lower demand for labor. The other consequences of the COVID-19 pandemic include reduction of job vacancies, productivity decline, and relocation of labor.^1^

With the beginning of the COVID-19 pandemic many migrant workers found themselves in uncertain and vulnerable situations. The pandemic economic crisis hit lots of industries which relied on migrant labor. As a result, many companies stopped hiring migrant workers, and many of them were thrust into reduction in working hours or unemployment. Some migrants found themselves in challenging situations because of being employed in sectors and occupations that do not allow for remote work but require personal contacts with coworkers and customers (Guadagno, 2020).

Despite adaptation of different policies and measures targeted at the migrant population in response to the COVID-19 situation (WHO, 2021), foreign workers experienced a severe negative impact of the pandemic crisis. Numerous studies reports that migrants are particularly exposed to the impacts of the COVID-19 pandemic due to their specific living conditions, insecure employment and precarious working conditions, inability to keep regular migration status because of job losses, and other obstacles that preclude them from the access to timely and appropriate health care (Guadagno, 2020; WHO, 2021). While being exposed to the higher risk of acquiring the virus, migrant communities suffer from numerous medical, economic, legal, and social negative socioeconomic outcomes of the COVID-19 (Clark et al., 2020; World Bank, 2020; WHO, 2021).

The main objective of the study is to identify migrants’ concerns and aspirations in a highly uncertain environment, which is the labor market during the COVID-19 pandemic. We focus on gender differences as some significant diversities were found when it comes to fears associated with pandemic (e.g., Fitzpatrick et al., 2020; Sasaki et al., 2022; Niño et al., 2021). E.g., Alsharawy et al., (2021) noticed that women reported greater fear and more negative expectations about health-related consequences of COVID-19 than men. However, women were more optimistic than men about the financial issues associated with the pandemic. Based on an extensive meta-analysis Metin et al. (2022) found a moderate and statistically significant effect of gender on COVID-19 related fear and anxiety. The authors also pointed out the role of cultural differences, as it turned out that females in Europe experienced higher fear and anxiety related to the pandemic than females in Middle East. We build on these setting and explore fears and hopes of men and women migrants connected with labor market participation. We also aim at qualitative investigation of interactions between identified topics.

The empirical analysis is based on 30 individual in-depth interviews with Ukrainian migrant workers. The interviews were conducted in February and March 2021 in Poland with participants who lived and worked in Poznań agglomeration.^2^ Poznan is one of the most popular destinations for migrant workers. The city was ranked second in terms of the number of registered declarations of entrusting work to a foreigner in 2020 (Statistics Poland, 2020). The situation on the local labor market was highly beneficial with a very low unemployment rate accompanied with high demand for both high-skilled and low-skilled workers. As a result, one of the main concerns of local employers has been the shortage of workers, generating sustained increased demand for labor. The circumstances began to change with the spread of the global COVID-19 pandemic at the end of the first quarter of 2020. The national lockdown introduced in March forced the closure of many companies. Epidemiological restrictions impacted the level of employment in local companies. As a result, the unemployment rate in the agglomeration began to rise and resulted in several concerns about the future of migrant workers. At that time, a massive number of Ukrainian citizens returned to their homeland because of uncertain employment prospects. Others decided to stay, sharing the hope for a fast recovery of the local economy. The paper contributes to the growing body of literature on the impact of the COVID-19 labor market shock on various demographic groups. Our unique methodological approach incorporates classical qualitative analysis with natural language processing methods (NLP), mainly sentiment analysis. The method allows for automatic extraction of sentiments from a given text corpora with specific algorithms (Hussein, 2018). NLP is not primarily used in economic research and is even rarer among the papers presenting field qualitative studies.^3^ However, it provides a powerful tool for in-depth text analysis with two key advantages: simplicity and statistical properties (Gonzalez-Hernandez et al., 2017; Wesslen, 2018) that are usually omitted in the classical IDI investigation. The dictionary-based sentiment analysis has the advantage of being empirically validated and robust in terms of findings (Asmussen & Møller, 2019; AIliev et al., 2014). In this paper, we take advantage of these new possibilities of text analysis. We believe that findings presented in this paper should be used and discussed in a broader context, as in a certain way they reflect results of government migration policies and labor market institutions functioning. According to the structural approach, these elements constitute the fundamental factors that could shape migrants’ decisions and migration patterns as presented in several influential contributions (e.g., Cassarino, 2004; Mohamed and Abdul-Talib (2020)).

The remainder of this paper is structured as follows. The second section reviews the relevant literature. The third next describes the qualitative data and research procedure. The fourth section presents the main findings. The last section summarizes and concludes with some recommendations.

## Literature Review

Several studies have documented migrant workers often experienced serious financial problems due to the reduced working hours or job losses caused by recent pandemic shock (e.g., Bhandari et al., 2021; Gama et al., 2022). It was estimated that over 75% of all OECD countries witnessed a significant increase in the unemployment rates among migrants in 2020 (OECD, 2021). Some evidence suggests that COVID-19 consequences were more severe for the older and lower-income immigrants (Bernstein et al., 2020; Gama et al., 2022). Another group that is particularly vulnerable to the long-term financial consequences resulting from the pandemic crisis are migrants with irregular status. This results from the prohibition of international trips accompanied by difficulties with traveling and returning to their home countries (Yovova, 2020).

A number of studies addressed various pandemic consequences with which non-nationals employed in the various economic industries and doing particular types of jobs are confronted. For example, research presented by Fassani and Mazza (2020) revealed that migrant workers who come from outside the EU tend to work in professions that are characterized by low teleworkability and high share of temporary contracts. Marschke et al. (2021) analyzed the impact of COVID-19 on the situation of fish workers in Thailand and Taiwan. Mitaritonna and Ragot (2020) focused on seasonal migrant workers employed in agriculture. Dempster and Zimmer (2020) as well as Singh and Singh (2020) examined the situation of migrants employed in the tourism and hospitality industry. 

Numerous studies have addressed psychological consequences induced by the COVID-19 pandemic which include anxiety (Surmai & Duff, 2022), increased stress level (Shabbir et al., 2022), depression (Shevlin et al., 2020), and lower psychological well-being (Vindegaard & Benros, 2020). Negative impact on psychological well-being could be more severe for migrant workers who cannot count on support from their families or friends in the host country, have limited social contacts or cannot travel to their home countries because of mobility restrictions (Kumar et al., 2020), and undefined registration status (Moawad & Andres 2020). Similarly, Spiritus-Beerden et al. (2021) found that the pandemic situation caused migrants’ and refugees’ mental health deterioration induced by increased perceived discrimination. Some groups of migrant population were confirmed to be particularly vulnerable to the pandemic's negative consequences. For example, Wang (2021) documented that children from migrant families had difficulties while dealing with the pandemic situation. Bhandari et al. (2021) provided evidence that immigrants experienced different negative emotions and thoughts caused by pandemic situations. Similar findings were presented in further quantitative and qualitative studies (e.g., Srivastava et al., 2021; Crouzet et al., 2022; Garrido et al., 2022).

Studies conducted in Poland (Brzozowski et al., 2020; EWL 2020, 2021; OTTO WorkForce, 2021) also widely confirm worsening migrant workers’ situation, including economic consequences (earnings decrease, number of tasks increase with no change in wages), working conditions (more procedures and restrictions), as well as the need to change industry accompanied by the decline of employment opportunities. For many migrant workers, the pandemic resulted in changing the place of work and/or one in four the place of living (EWL 2020, 2021).

Some reports confirm that the COVID-19 pandemic led to the increasing number of racism, xenophobia, and discrimination cases in many countries, also in Poland (Pietrzak n.d.). For example, the non-for-profit organization *Nigdy Więcej* (eng. Never Again) has documented 30 episodes of hate speech, assault, and unfounded blaming of migrants for spreading the virus (*Wirus nienawiści: „Brunatna Księga” czasu epidemii (eng. The Hate Virus: The Brown Book of the Epidemic)* 2020)*.* Increased aversion to foreigners can be an additional obstacle for them to have access to the healthcare system (Guadagno, 2020) or find legal employment in times of the pandemic (Pietrzak n.d.).

All the above-mentioned factors combined with lack of access to complete and accurate information can lead to increased migrants’ sense of insecurity (Magdziarz et al., 2021) and affect their psychological well-being. Empirical studies suggest that one of the most often reported feelings is fear about the future, including employment prospects. For example, stress related to the future was mentioned as a major challenge of living during the pandemic (Як cпpaви? How are You? Jak się masz? 2020). Among the surveyed temporary workers from Ukraine, more than half (57%) were afraid of losing their jobs (OTTO WorkForce, 2021). Loneliness resulting from limited personal contacts and traveling restrictions was also a reason for psychological problems (Brzozowski et al., 2020).

The majority of studies conducted so far have focused more on mental health and psychological problems of migrants (Table 1), and their research design did not take into account their possible aspirations and hopes. Therefore, the conclusions presented in our paper constitute an essential contribution to the literature on the situation of migrant workers during the pandemic.

**Table 1:** The summary of research on migrants concerns which result from the pandemic situation

Author(s)	Identified concerns and psychological effects
Kumar et al. (2020)	Anxiety and depressive symptoms
Spiritus-Beerden et al. (2021)	Deterioration in mental condition
Bhandari et al. (2021)	Worried feelings and thoughts, health problems, financial hardship, family concerns, confusion, and anxiety associated with being infected by COVID-19 virus
Srivastava et al. (2021)	Major stressors: financial crisis, lack of social support, unavailability of food, inability to continue education, inability to pay house rent, uncertainty of the quarantine period, psychological stressors (uncertainty of the duration of lockdown, uncertainty of jobs and the future, fear of the spread of the pandemic)
Crouzet et al. (2022)	Feeling of being neglected by the authorities, deteriorating health, delayed administrative procedures, financial difficulties, difficulties shopping for basic necessities
Garrido et al. (2022)	Decrease in psychological well-being
*Як cпpaви? How are You? Jak się masz?* (2020)	Concerns related to the possible change of employment conditions, job loss, stress related to the future, lack of employment, health concerns, lower quality of life
Brzozowski et al. (2020)	Loneliness, confusion, psychological problems, and fear
OTTO WorkForce (2021)	Fear of losing a job
Magdziarz et al. (2021)	Lack of a sense of residence security, problems with receiving up-to-date and understandable messages regarding legal situation or access to healthcare

Source: own elaborations based on the literature review

## Qualitative Data and Research Procedure

The qualitative data were collected in February and March, 2021 from 30 Ukrainians using individual in-depth interviews (IDI) technique. Interviewees were purposively selected migrants who had resided in Poland for at least 1 year before the pandemic started.^4^ Initially, interviewers used their social networks to find participants. Then each interviewee was asked to recommend the further two persons who fit the selection criteria. During the research, we ensured application of procedures and quality standards set out in the Quality Control Program introduced by the Organization of Opinion and Market Research Companies in Poland and General Data Protection Regulation (GDPR). A letter explaining the purpose, approach, and dissemination strategy of the research was prepared and shared with all the IDI participants. A clear verbal explanation was also provided to each interviewee. IDI transcripts were anonymized and details that could be used to identify participants were removed.

All study participants had a paid job or were actively looking for employment at the time of participation in the study. The spatial scope of the study was Poznan agglomeration^5^ which includes the city of Poznań and 17 surrounding counties situated in Western Poland. The final research sample consisted of 15 men and 15 women. The participants were aged between 21 and 52 years.

Of all participants, 13 migrants had tertiary education. Of the 30 respondents in the study, 7 (23.33%) were employed in construction. The others had jobs in accommodation and food services (6 participants, 20%), transport and storage (4 participants, 13.33%), trade (4 participants, 13.33%), manufacturing (3 participants, 10%). The remaining 6 participants were employed in other sectors, mostly services. A total of 66.6% of interviewees performed physically demanding jobs and 33.3% were employed in intellectual jobs. Due to pandemic restrictions and health security reasons half of the interviews were conducted online (via Skype). Each interviewee could decide which language one preferred to speak with the interviewer. Therefore, most of the interviews were conducted in Ukrainian or Russian, and one interview was conducted in Polish. The interview lasted between 24 and 71 min. Face-to-face interviews were audio recorded. Online interviews were video recorded.

The IDI questionnaire was semi-structured. The general topics and specific issues covered during the interviews are presented in Table 2. At the beginning of the meeting an interviewer showed the participant a piece of paper with a timeline drawn on it. It consisted of three points: “March, 2019” that is 1 year before the pandemic outbreak in Poland, “March, 2020” (the pandemic outbreak in Poland), and “Today”. A participant was asked to recall all the situations and events relevant to one's employment and life situation. Then, a participant was asked to think of her/his current situation on the labor market and plans for the future. An interviewee could make notes on the template and freely use them during the interview. In case of an online interview, the template was sent via the Internet and an interviewee could make notes on her/his electronic device.

**Table 2:** The general structure of the interview

General topic	Issues
Employment history	• *purposes of arrival to Poland* • *employment history*
Situation before the COVID-19 pandemic	• *career goals* • *employment situation*
Situation during the COVID-19 pandemic	• *current career goals* • *impact of the COVID-19 pandemic on migrants’ life and employment situation* • *risks and changes on the labor market* • *treatment by employers* • *employers’ expectations* • *perceptions on security and stability*
Plans for the future	• *future career goals, plans and perspectives* • *(subjective) chances of pursuing career goals and aspirations* • *limitations and barriers that may influence the achievement of career goals*

Source: own elaborations

The interviews’ transcripts were translated into English and in the first step of the research procedure split into single words (tokens) and analyzed. The men’ interviews consist of 8288 unique words. In turn, the women’ interviews consist of 10,938 unique words.

**Fig. 1:**
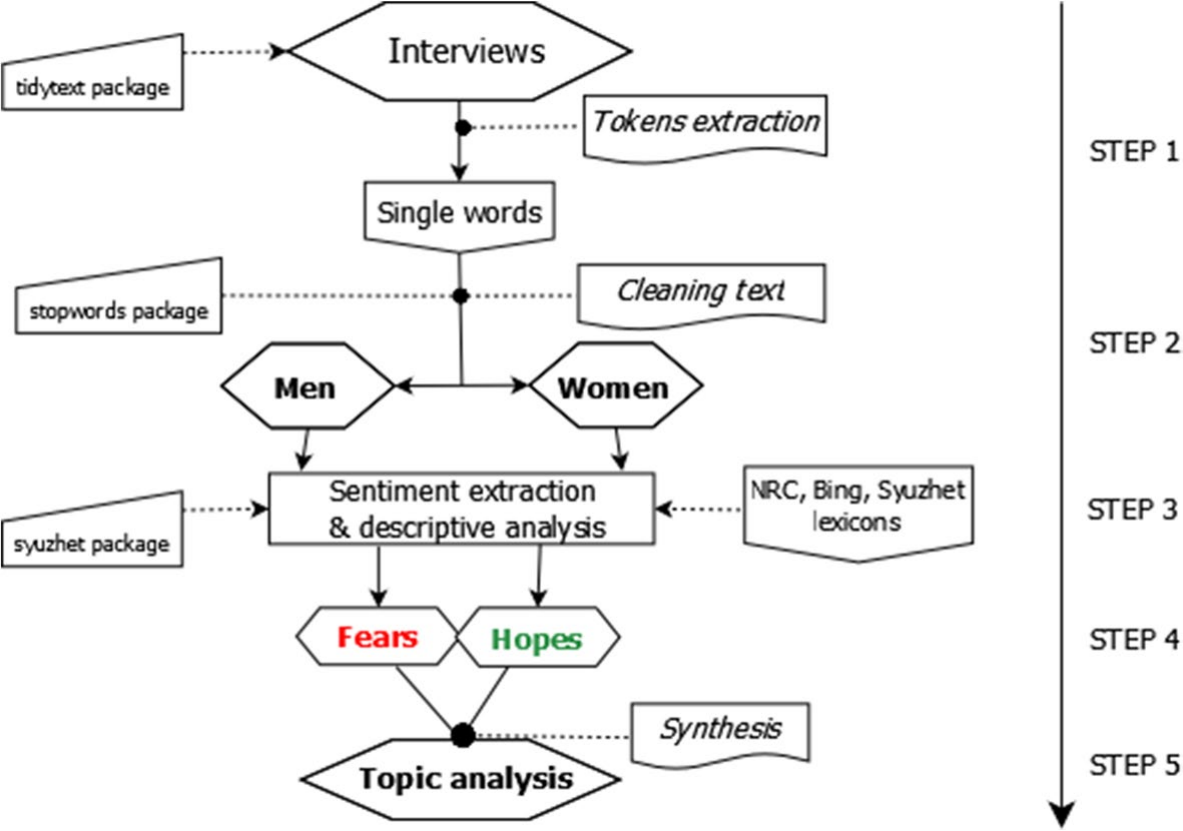
The schema of research procedure. Source: own elaborations

Then the corpus was cleared away of the sentences spoken by the interviewers. The interviews were also cleaned of so-called stop words. Stop words are words that are not useful for an analysis, typically extremely common words such as “the”, “of”, “to”. In this procedure we applied R package *stop_words* (Benoit et al., 2021) that consists of 1152 of such unnecessary phrases. The sentiment analysis was applied on the cleaned data with the excellent *tidytext* R package by Silge and Robinson (2016) and *syuzhet* by Jockers (2015). Finally, we manually extracted topics and associated them with given sentiment words. The general schema of the research procedure is shown in Fig. 1.

## Sentiment Analysis

### Descriptive Analysis

In this research step we adopted sentiment analysis which we used to identify fears and hopes that share migrant workers from Ukraine. The interviews were splitted depending on gender as some significant differences in sentiment could be observed between men and women narrations (e.g., Thelwall, 2018; De Amicis et al., 2021).

We used two lexicons (Bing and Syuzhet) to extract positive and negative words and calculate sentiment scores for the interviews. These two particular dictionaries were applied for comparative purposes so one can observe the potential differences in extracted words and sentiment scores between them. To take out certain emotions we used the NRC emotion lexicon. Some general notes on applied dictionaries are juxtaposed in Table 3.

**Table 3:** The lexicons used in the sentiment analysis

Lexicon	Positive words	Negative words	Total words	Scale	Authors
Bing	2006	4783	6789	2 levels (positive and negative)	Hu and Liu (2004)
Syuzhet	3587	7161	10,748	2 levels (positive and negative classified on the scale from − 1 to 1 with 16 grades)	Jockers and Thalken (2014)
NRC Emotion	2312	3324	5555	10 levels (fear, joy, anger, sadness, anticipation, disgust, surprise, trust, positive, negative)	Mohammad and Turney (2013)

Source: own elaborations

The Bing lexicon is a general-purpose English sentiment lexicon that categorizes words in a binary fashion, either positive or negative (Hu & Liu, 2004). In turn, NRC Emotion categorizes words in 8 basic emotions and two sentiments (either positive or negative) in the same way as Bing. The calculation of sentiment score for these two lexicons is done according to the simple formula:

$$\left({\varvec{n}}{\varvec{u}}{\varvec{m}}{\varvec{b}}{\varvec{e}}{\varvec{r}}\boldsymbol{ }{\varvec{o}}{\varvec{f}}\boldsymbol{ }{\varvec{p}}{\varvec{o}}{\varvec{s}}{\varvec{i}}{\varvec{t}}{\varvec{i}}{\varvec{v}}{\varvec{e}}\boldsymbol{ }{\varvec{w}}{\varvec{o}}{\varvec{r}}{\varvec{d}}{\varvec{s}}-\boldsymbol{ }{\varvec{n}}{\varvec{u}}{\varvec{m}}{\varvec{b}}{\varvec{e}}{\varvec{r}}\boldsymbol{ }{\varvec{o}}{\varvec{f}}\boldsymbol{ }{\varvec{n}}{\varvec{e}}{\varvec{g}}{\varvec{a}}{\varvec{t}}{\varvec{i}}{\varvec{v}}{\varvec{e}}\boldsymbol{ }{\varvec{w}}{\varvec{o}}{\varvec{r}}{\varvec{d}}{\varvec{s}}\right)/{\varvec{t}}{\varvec{o}}{\varvec{t}}{\varvec{a}}{\varvec{l}}\boldsymbol{ }{\varvec{n}}{\varvec{u}}{\varvec{m}}{\varvec{b}}{\varvec{e}}{\varvec{r}}\boldsymbol{ }{\varvec{o}}{\varvec{f}}\boldsymbol{ }{\varvec{w}}{\varvec{o}}{\varvec{r}}{\varvec{d}}{\varvec{s}}$$

The basic Syuzhet lexicon was developed in the Nebraska Literary Lab (Naldi, 2019) and calculates sentiment in a different way. It contains 10,748 words which are assigned a score of − 1 to 1 with 16 gradients. In order to produce a sentiment score, Syuzhet assigns each word a score from the range given above (Jockers and Thalken, 2014).

Some controversial issues for these dictionaries regard mainly the classification of words. E.g., NRC Emotion may produce higher sentiment scores as many neutral words are classified as positive. In turn, Syuzhet classifies some neutral words as positive which may also disturb the assessment of text (Stephen et al., 2020; Sonkin, 2021). Including three dictionaries in the analysis will help us to prevent potential misclassification problems reported in the literature and broaden the scope of the analysis.

**Fig. 2:**
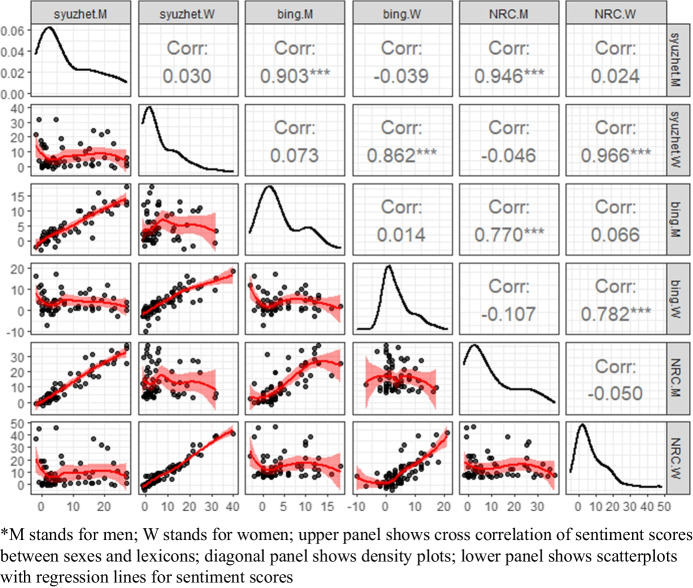
Scatterplot matrix for sentiment scores depending on sex and lexicon. Source: own computations

In Fig. 2 the correlations between obtained sentiment scores are presented so one can compare the results obtained with a given dictionary.

In general, the strong and significant correlation is observed between sentiment scores and lexicons within the same sex (e.g., Syuzhet and NRC for women have 0.966 correlation and for men 0.946). It means that the dictionaries captured emotional valence in the interviews in a similar way. However, NRC and Bing produced slightly lower cross-correlation scores than NRC and Syuzhet lexicons. In turn, scores between sexes are not correlated even within the same lexicon. It provides evidence for significant differences between men and women interviews in terms of sentiment and corresponds with the literature.

**Fig. 3:**
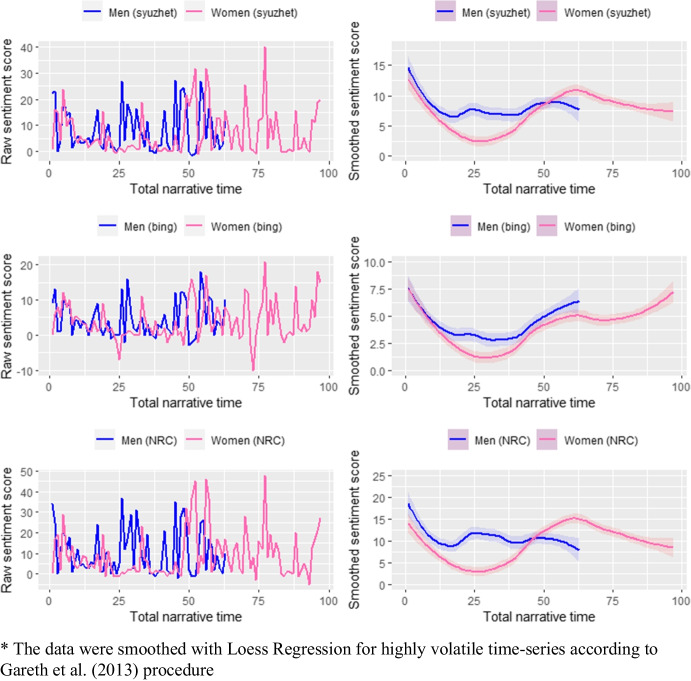
The sentiment values for sexes and lexicons (raw and smoothed)*. Source: own calculations

In the next figure (Fig. 3) we plotted raw and smoothed sentiment scores for men and women. Because of some differences in the lexicons’ design and scale, the scores also differ.

In general, the computed sentiment scores were lower for women. It means that these interviews were characterized by more negative emotional valence. There are also larger differences between maximum and minimum values in women’ interviews which indicate bigger fluctuations of sentiment compared to men. Likewise, the standard deviations are on average slightly higher in women interviews. It can be observed within all lexicons. The smoothed trajectories of interviews are quite similar in all dictionaries. According to NRC and Syuzhet, men’s sentiment scores fall below women’s at the end of narrations. This decrease is not confirmed by the Bing lexicon which produced opposite results at the end of the narration line.

### *Exploring Fears and Hopes*

In the fourth step of the research procedure, we focused on extraction and classification of specific concerns (fears) and expectations (hopes) that appear in narrations. The lexicon that directly incorporates these emotions (fear and anticipation) is NRC Emotions. However, we also made an attempt to extract fears and hopes from positive and negative words as classified by Bing and Syuzhet dictionaries.

**Fig. 4:**
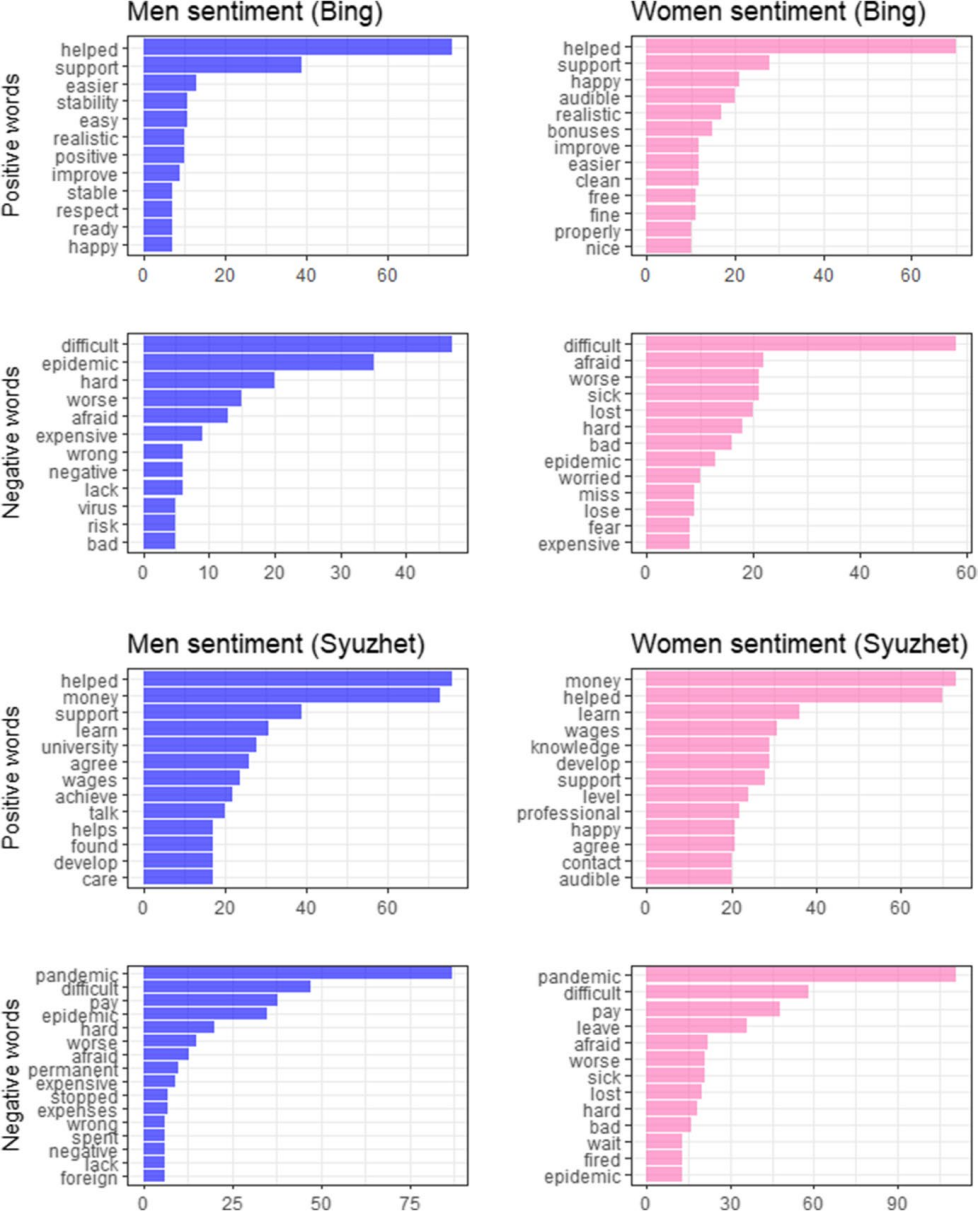
Top positive and negative words in interviews. Source: own calculations

Bing lexicon identified 194 positive words for women and 148 for men. In turn, there were 202 negative words in women interviews and 157 in men. According to the Syuzhet lexicon there were 449 positive words in women narration and 376 in men. Negative collocations were identified among 289 words in women’ interviews and among 253 words in men’. The inverse proportion of positive and negative words between the NRC and the two other dictionaries may be caused by the differences in lexicons’ design (specific emotions vs two level sentiment). Moreover, it is worth adding that Syuzhet lexicon classifies as positive words that should be classified as neutral, which was previously pointed out by Sonkin (2021). In Fig. 4, we plotted top positive and top negative words with frequencies divided by lexicon and sex.

**Fig. 5:**
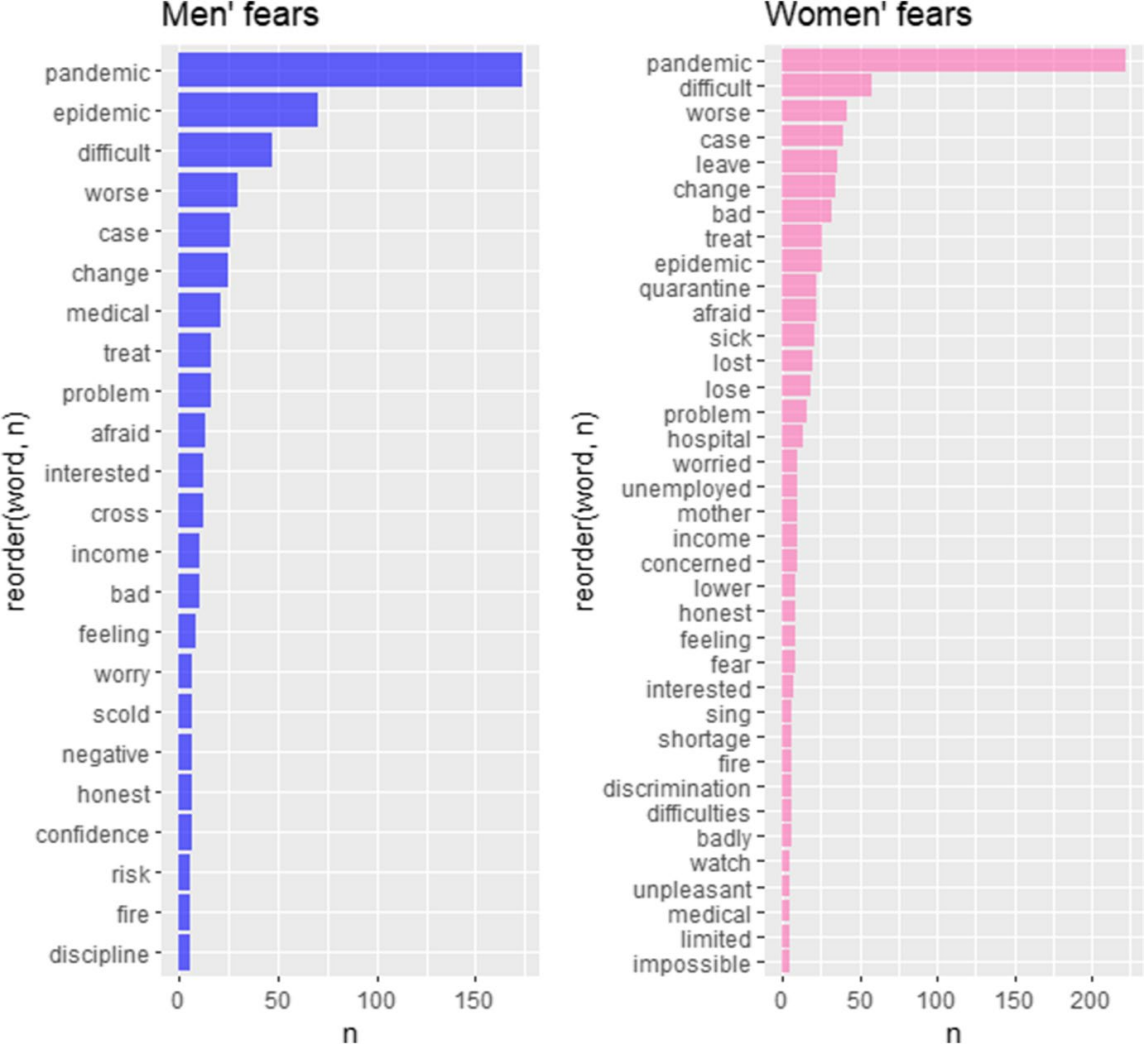
The most frequent men’s and women’s fears (NRC emotions). Source: own calculations

As previously mentioned, the respondents’ fears were extracted with the NRC lexicon, the only dictionary in which words are connected with basic emotions. Two sentiment values were filtered: “fear” and “sadness” (Fig. 5).

On a basis of negative words (Bing, Syuzhet) and fears (NRC) drawn from the interviews, we identified thematic areas that these words may be associated with. Some of them are common to both men and women while others are more specific for a given group. In such a case we assigned the topics to the sex where topic words were significantly more frequent. If the words appeared in both men and women narrations with similar frequency, we assumed the topic is common. In Table 4 we presented topics with exemplary words by gender; in Table 5 there are some quotations that link to the extracted topics.

**Table 4:** The extracted topics associated with fears

Topic	Exemplary words	Men/Women
Pandemic issues	*quarantine, infection, sick*	Both
Medical care and health	*hospital, die, sick, cancer, death, fever*	Women
Job instability	*fire, risk, unemployed, losing, quit*	Both
Financial situation	*bankrupt, income, cash, pay, expensive*	Men
Uncertain life change	*leave, lost, shortage, difficulties, lower, lack*	Both
Discrimination	*scold, stranger, unfriendly, aggressive*	Women
Social status decline	*degradation, fallen, withdrawn, detorierted*	Men
Feelings inducing fears	*panic, scared, anger, nervous,*	Women
Other psychological concerns and barriers	*worried, impossible, limited, refuse, stress, lie, obstacles, drop*	Both

Source: own elaborations on the basis of extracted fears

**Table 5:** Exemplary quotations for topics associated with fears

Topic	Illustrative quotations
Pandemic issues	*(…)because of the pandemic, you don't know what will happen tomorrow*
Medical care and health	*You don't know whether you will wake up or not. (…) why a person wakes up one day, happy and healthy, and the next day finds out they have cancer*
Job instability	*The service sector is collapsing, some hotel chains, cafes, catering—many people (…) I talked to said they had lost their jobs*
Financial situation	*Wages have also decreased. People will go to any job just to be employed*
Uncertain life change	*(…) when you arrive, you do not need to feel lost. You need to know that you did not leave the country because you had no choice* *There are always difficulties, that is life. (…) you must rethink your plans*
Discrimination	*I felt an aggressive attitude (…). This was openly expressed in words*
Social status decline	*There was less work, wages decreased too, and there was a deterioration in [my] financial standing (…). It all began in March*
Feelings inducing fears	*(…)there was panic, we were locked in our houses, we were afraid of every person*
Other psychological concerns and barriers	*I worried about what other people would think of me (…)* *I had huge mood swings, I wanted to drop everything and leave (…)*

Source: own elaborations

*Pandemic issues* are the leading topic across men and women interviews; however, the women pay much more attention to the details connected with this area that leads to the additional fear connected with the *medical care system and health*. In turn, men are more afraid of worsening of the *financial situation* and, as an implication, *decline in their social status*. The presented differences can be explained by the traditional model of economic migration from Ukraine to Poland, which consisted in the fact that mainly men left in search of better job and decent earnings. In Ukrainian patriarchal society, it is the man who is perceived as the main breadwinner of the family; hence, for male migrants the pandemic appears to be a threat that makes it impossible to fulfill the basic role in the family. *Job instability* topic is of a high interest in both types of interviews which is not surprising as pandemic affected mostly economic branches and professions which are typically performed by foreigners. *Psychological concerns and barriers* induce difficult feelings, but also these feelings may trigger psychological concerns.

**Fig. 6:**
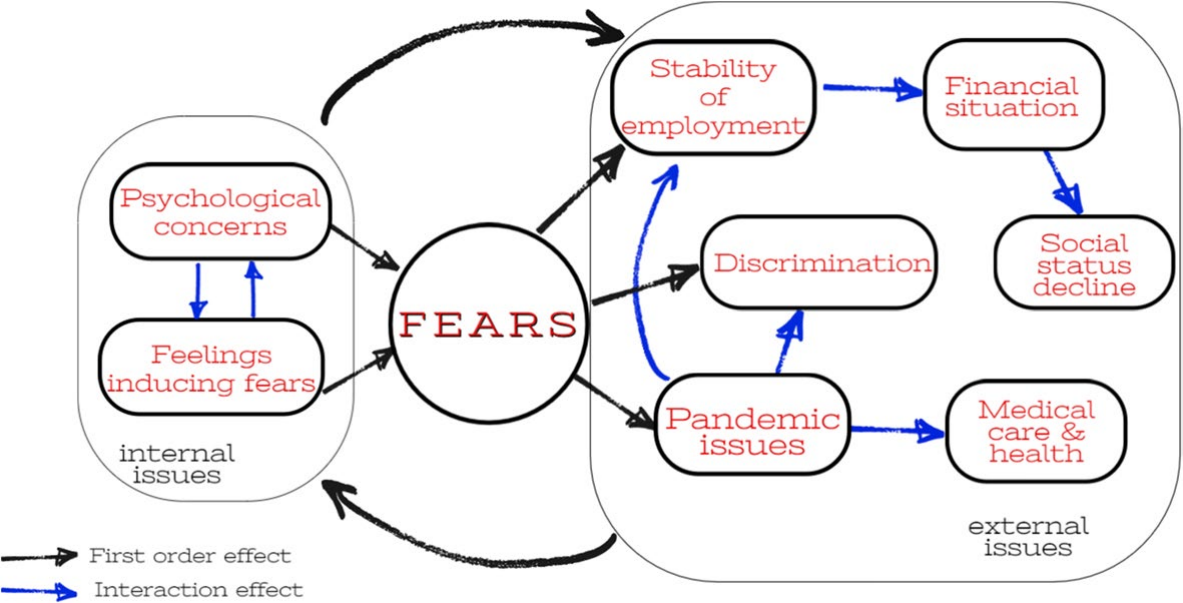
Graph of topics connected with fears. Source: own elaborations

Finally, we visualized the thematic areas linked to fears in Fig. 6. The connection between concerns is rather straightforward. The arrows indicate the direction of the relationship. There are first-order fears and interaction fears. While the former is a basic, low-level concern, the latter is usually the derivative (expansion) of these low-level problems. For instance, *pandemic issues* directly induce fears about *medical care and health*, *stability of employment*, and *discrimination*. In turn, stability of employment triggers further fear connected with *the financial situation.* The latter leads to another concern which is *status downgrade.* These are examples of interaction effects*.* The pandemic directly induced topics connected with medical care, stability of employment, and discrimination. The discrimination may reversibly induce problems with finding employment also in rich European economies as pointed out by Falck (2021).

The identified topics connected with fears could be divided into two blocks. The first block covers internal issues, and the second block refers to external issues. Internal issues include topics connected with individual personality type, and external issues are the topics associated with economic conditions during pandemic. The interaction between these blocks may produce the self-winding spiral of fears, i.e., internal topics may induce external problems and vice versa external topics may boost internal concerns.

**Fig. 7:**
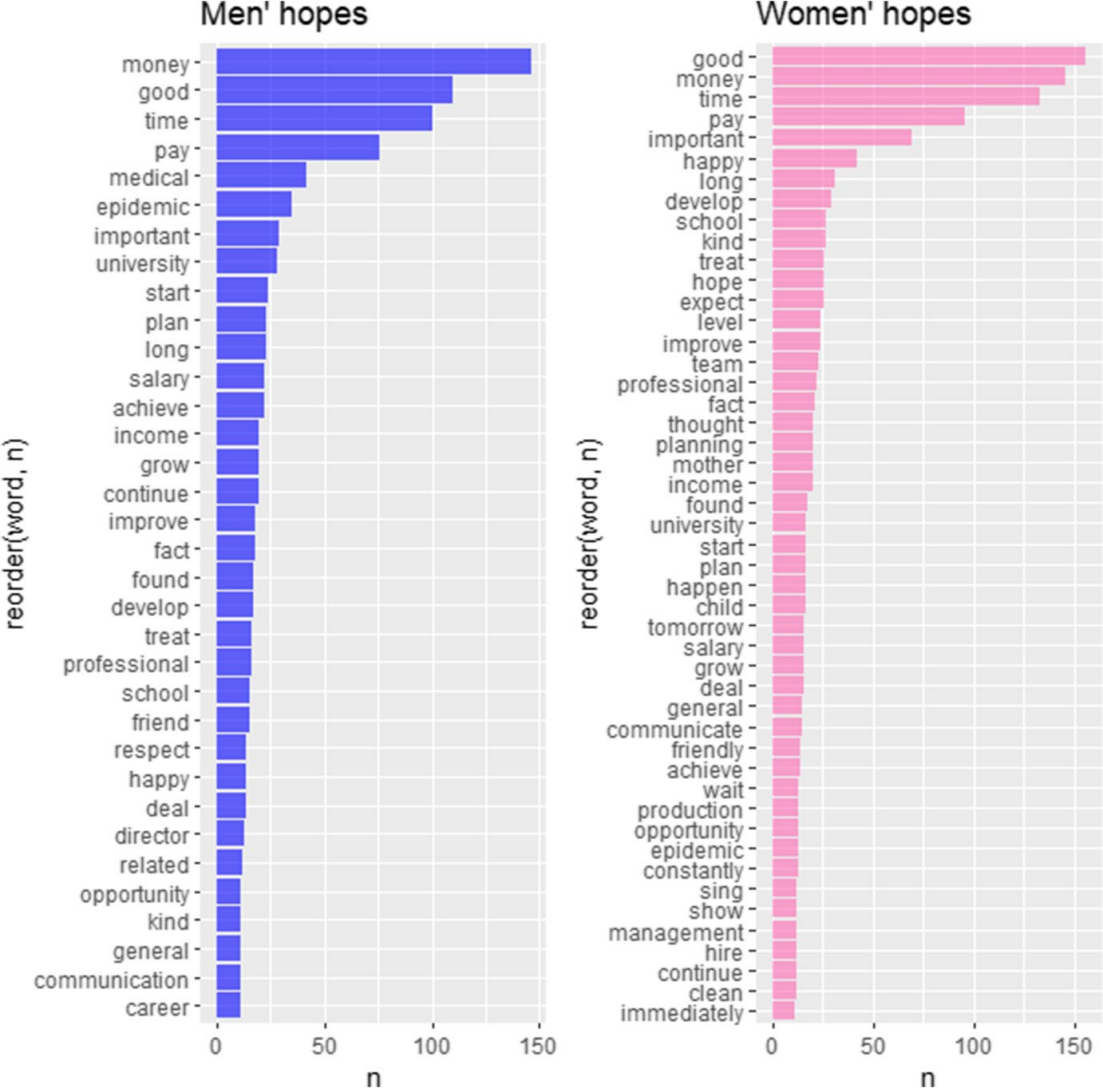
The most frequent men’s and women’s hopes (NRC emotions). Source: own calculations

A similar procedure was applied to extract respondents’ hopes. These were also extracted with the NRC lexicon. Two sentiment values were filtered in this case: ‘anticipation’ and ‘trust’. The results are presented in Fig. 7.

In Table 6 we presented extracted topics together with an indication of the respondents’ group (men or women) and exemplary words, while Table 7 presents some illustrative quotations from the interviews.

**Table 6:** The extracted topics associated with hopes

Topic	Exemplary words	Men/Women
Pandemic issues and health	*medical, epidemic, risk*	Men
Family and childcare	*school, mother, child, father*	Women
Financial situation	*money, pay, salary, pay, income, cash, compensate*	Both
Professional and personal growth	*develop, start, improve, start, achieve, opportunity*	Both
Learning	*knowledge, learn, university, learning*	Both
Relationships	*treat, respect, team, friend, communication, communicate, help, support*	Both
Feelings inducing aspirations	*kind, happy, moral, good, stable, hope, expect*	Both

Source: own elaborations on the basis of extracted hopes

**Table 7:** Exemplary quotations for topics associated with hopes

Topic	Illustrative quotations
Pandemic issues and health	*(…) I am also a student and I have to combine work and studies. Thanks to the pandemic (…) I do not have to go anywhere—neither to study nor to work. (…) Therefore, my life and my work have been positively affected*
Family and childcare	*(…)my mother and two grandmothers, they always call me and always ask if I'm okay. Whenever I need some help, I can get in touch with them and they help me a lot*
Financial situation	*There is financial support, which I need for a certain project or for survival, because things have happened differently*
Professional and personal growth	*And in terms of personal qualities, I am developing leadership qualities because working with people requires patience*
Learning	*During this pandemic, when you don't know what will happen tomorrow, I personally started to live faster, to try everything faster, to learn faster, to change*
Relationships	*(…) he helped me obtain my first residence card. I became friends with him*
Feelings inducing aspirations	*I hope to create my own company in the future and cooperate with my current boss. That is my goal*

Source: own elaborations

**Fig. 8:**
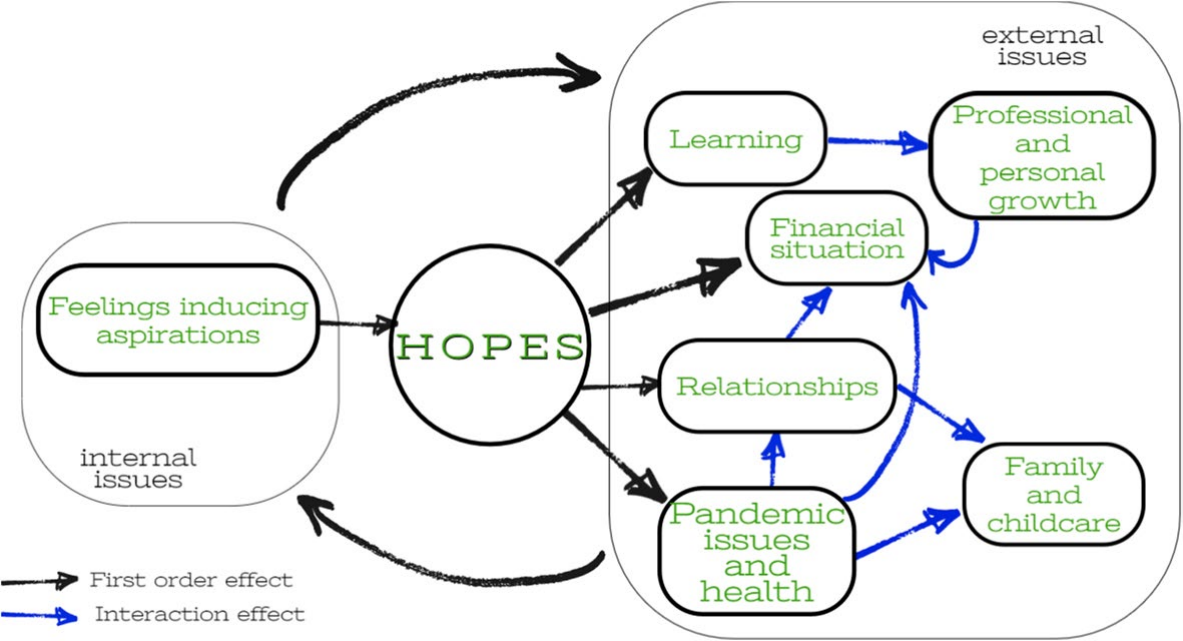
Graph of topics connected with hopes. Source: own elaborations

In case of hopes, the majority of identified topics were common for both male and female participants. The most important topic related to hopes covered *Financial issues*. It is closely associated with another two relevant topics, namely *Personal and professional growth* and *Learning*. These could be seen as migrants’ readiness to invest in individual human capital to make possible the improvement of financial and life situations. Another common topic, *Relationships*, shows the importance of personal and professional contacts in dealing with a pandemic situation and accompanying problems. Figure 8 presents a visualization of the identified topics connected with hopes.

Similar to the fears analysis, we observed several interactions. These include, for example, the mutual effect between *Pandemic issues and health* and *Relationships*. Safety regulations and restrictions have significantly reduced the opportunities for integration and adaptation of migrant workers. At the same time, support from Polish colleagues and friends, as well as compatriots, could help to deal with isolation and loneliness. A very similar interaction effect can be observed in the case of *Financial situation*. Economic shock caused slowing economic activity and reduced demand for migrant labor. As a result, migrant workers had to work reduced hours or even lost their jobs. Many of them found themselves trapped in destination countries with the lack of financial resources and no access to social protection. In turn, a worse financial situation is a serious barrier to paying for private medical care services and providing safe housing conditions, which could lead to lower health safety.

## Conclusions and Discussion

In the paper we aimed at identifying fears and hopes of migrants’ workers during the economic shock, which is COVID-19 pandemic. We used an innovative research approach which combined natural language processing techniques with classical qualitative analysis. We employed sentiment analysis to analyze 30 individual in-depth interviews with Ukrainian economic immigrants residing in Poland. We also extracted topics that are associated with migrants’ concerns and ambitions. The adopted methodology allowed us to apply reliable in-depth data exploration procedures.

Our findings overlap in some points with previous studies, complement it in some points, and contradict in others. For example, similarly to the research conducted by Guadagno (2020), we confirmed that discrimination may be an important problem for migrant workers, especially during economic shock. However, we observed that the topic was much more vital in women’ interviews. Results confirm that for Ukrainian migrant workers, the most serious pandemic effects were associated with economic consequences accompanied by discrimination and employment stability. While similar observations were documented in previous contributions (e.g., OTTO Workforce, 2021), our findings also capture interactions between specific weaknesses of the Polish national system of migrants’ protection. From this point of view, suppressing first-order effects (fears) should also reduce interaction effects (fears) without direct intervention. Such an approach could be extremely useful from the point of view of the policy solutions implementation.

We identified clear differences between men and women interviews in terms of descriptive, as well as sentiment and topic analysis. In general women interviews are longer, contain more diversified vocabulary, have lower average sentiment score and larger standard deviation. The identified thematic areas share some similarities, but we also observed contrasts. The topics of *the medical system, health care, and family* are more distinctive for women’s interviews, therefore, the topics associated with household safety in the broad sense. In turn, *financial situation* and *social status* are rather the characteristics of men’s narrations. These areas are strictly connected with money and economic stability of household. The gender differences could be explained with traditional roles of Ukrainian migrants—men are those who bring money; women are those who take care of family well-being.

The results indicate that *stability of employment* is a crucial fear of migrant workers that affects their quality of life and well-being, as it may induce further fears and problems connected with financial problems and social status.

The findings reveal also that while men and women experience rather different fears associated with the pandemic situation, both groups share the majority of hopes. We also identified some overlapping topics—the topics that are associated with both fears and hopes across men and women narrations. These could be perceived as key elements of migrants’ workers’ everyday lives during the pandemic era. The most important topic is *Financial issues,* which is the derivative of *Stability of employment* fear, as well as key migrants’ hope to which majority of other ambitions lead.

The paper contributes to the existing research in three ways. Firstly, the study provides new insights into the economic migrants’ situation on the labor market in Poland during the pandemic times. Secondly, our findings point to the solutions that may support migrants on the labor market during economic shock. In this regard, we believe that the migrants’ fears could be significantly reduced by providing wide informational support focusing on the most essential areas (healthcare, access to vaccinations, social support, traveling restrictions, legalization of residence, and work, etc.).

Governments should ensure that accurate and up-to-date information about local epidemiologic situations is available and accessible to all migrants, regardless of their status. This can include translating information into multiple languages and disseminating it through various channels, such as social media, radio, and community organizations. These recommendations also go along with findings presented by Magdziarz et al. (2021) who proved that lack of information increases stress and uncertainty among migrants, and the pandemic induces these feelings. Active steps should be taken to ensure that migrants have access to healthcare services without fear of being reported to immigration authorities. Some other important actions should consider psychological support, discrimination prevention, as well as measures to prevent the spread of disinformation about migrants. The latter issue can be an additional factor that causes rising migrants’ feeling of insecurity. Local governments should work with community leaders and organizations to disseminate accurate information about COVID-19 and to dispel any disinformation that may be circulating. Community leaders and organizations can help build trust and credibility with their communities and can play an important role in promoting public health messages. Sufficient support should be provided for vulnerable populations, such as undocumented migrants, who may be particularly vulnerable to disinformation and may be hesitant to seek healthcare services due to fear of deportation. This can include providing access to healthcare services without requiring documentation, as well as financial and social support to help mitigate the economic impact of the pandemic.

Finally, our paper contributes to the emerging strand of literature as it jointly explores the concerns and ambitions of economic immigrants, in contrast to the majority of previous contributions that focused mainly on threats and psychological problems that migrants have to cope with (e.g., Kumar et al., 2020; Srivastava et al., 2021; Crouzet et al., 2022; Garrido et al., 2022).

Further exploration of migrant workers’ fears and hopes would be a valuable source of information about domains in which most activities should be focused during the times of economic shocks. The relationships provided in this paper have to be verified in future studies based on more extensive (probably quantitative) data. The findings will be valuable for developing and implementing more effective local policies.

## Footnotes

1 See Brodeur et al., (2020) for in-depth literature review.

2 The city is situated in Western Poland. It is the fifth largest city in the country in terms of population (536,438 inhabitants) and sixth in terms of area (262 km^2^). In the whole agglomerations live 1 million inhabitants in an area of 3000 km^2^.

3 A large number of scientific applications regard medical research (e.g., Sheikhalishahi et al., 2019; Wu et al. 2023). Recently, there is a growing body of literature on bibliometric analysis with the use of NLP-based methods, also in the area of migration (e.g., Pisarevskaya et al., 2020). Other applications involve social media analysis (e.g., Patton et al., 2020). It can be also applied for some problems in economics, mostly for finance and marketing issues (e.g., Proellochs & Feuerriegel, 2021).

4 March, 2020.

5 It is the fifth largest city in Poland in terms of population (536,438 inhabitants) and sixth in terms of area (262 km^2^). In the whole agglomerations live 1 million inhabitants in an area of 3000 km^2^. It is also the third most popular destination for migrant workers in Poland.
